# A Multidisciplinary Approach: Management and Rehabilitation of Children With Pediatric Post-COVID-19 Condition

**DOI:** 10.1097/INF.0000000000004408

**Published:** 2024-05-29

**Authors:** Lieke Noij, Suzanne Terheggen-Lagro, Eefje Muselaers, Elizabeth Whittaker, Justine Gosling, Caroline Brackel, Kim Oostrom, Mattijs Alsem

**Affiliations:** * From the Department of Pediatric Pulmonology and Allergy, Emma Children’s Hospital, Amsterdam University Medical Centers; † Department of Rehabilitation Medicine, Amsterdam Movement Sciences, Amsterdam, The Netherlands; ‡ Department of Pediatric Infectious Diseases, Imperial College Healthcare NHS Trust; § Section of Pediatric Infectious Diseases, Imperial College, London, United Kingdom; ¶ WHO Regional Office for Europe, Copenhagen, Denmark; ‖ Department of Pediatrics, Tergooi MC, Hilversum, The Netherlands; ** Department of Child and Adolescent Psychiatry and Psychosocial Care, Amsterdam Reproduction & Development, Amsterdam University Medical Centers, Amsterdam; †† Department of Rehabilitation, Physical Therapy Science and Sports, University Medical Center Utrecht, Utrecht, The Netherlands.

**Keywords:** pediatric post-COVID condition, long COVID, management, rehabilitation, children

## Abstract

Post-COVID-19 condition in children is a still largely unknown syndrome with a diverse pattern of symptoms, which can have a major impact on daily life. Currently, there are no evidence-based proven treatments, and the focus is on symptom management and recovery of daily functioning. A multidisciplinary, tailored approach is recommended, with attention to energy management and activity building, where the main goal should be a return to baseline levels of cognitive, physical and social activity.

Infections with SARS-CoV-2, also known as coronavirus disease 2019 (COVID-19), are relatively mild in children, with only a small percentage leading to hospital admissions.^1,2^ There is however, a subgroup of children who suffer from long-term symptoms and impairments in neurocognitive, psychosocial and physical functioning with decreased societal participation after infection with SARS-CoV-2.^3–5^ This condition, known as Long COVID or postacute sequelae of SARS-CoV-2 infection in adults, is in children commonly referred to as pediatric post-COVID-19 condition (PPCC), and was defined by the World Health Organization as “symptoms lasting at least 2 months, which initially occurred within 3 months of acute COVID-19, which cannot be explained by an alternative diagnosis.”^6^ The pathophysiology of PPCC is poorly characterized and not yet fully understood,^1,7^ however, the following possible underlying mechanisms for post-COVID-19 syndrome in adults have been described: immune dysregulation (eg, due to viral persistence), microbiota dysbiosis, autoimmunity and immune priming, clotting and endothelial abnormalities, and/or dysfunctional neurological signaling.^8–11^ The reported prevalence of PPCC has a wide range of 1.6% to 67%,^12,13^ which could be due to differences in study populations (hospitalized vs. nonhospitalized), different follow-up approaches, or lack of consensus on PPCC definitions. The most common PPCC symptoms include fatigue, mood symptoms, headache, cognitive difficulties, respiratory symptoms and loss of smell.^14,15^

Because the precise pathophysiology of PPCC remains uncertain, there are currently no established treatment strategies targeting etiological mechanisms available. Therefore, the current approach remains centered on symptom management and a gradual return to activities. A team of American healthcare professionals developed a Consensus Guidance Statement on PAS-C in Children and Adolescents^16^ to assist the primary care physician and initial specialty evaluations for children and adolescents with PPCC. Additionally, a review by Yonts et al^17^ on the current evaluation and management strategies in PPCC showed that children benefit from an individualized, multidisciplinary approach, with a focus on physical and psychological rehabilitation, where the main goal should be a return to baseline levels of cognitive, physical and social activity.

Based on available evidence for children and adults^18–24^ and our experiences in the care for children with PPCC,^25^ as well as our expertise in children and adolescents with other chronic (fatigue) conditions, we formulated practical guidance for the treatment of children PPCC by a multidisciplinary team. This guidance was established through a collaboration of a diverse group of healthcare professionals (eg, pediatric pulmonologists, pediatric rehabilitation physicians, pediatric immunologists, pediatric psychologists, pediatric physio- and ergotherapists, dieticians) from the Netherlands and the United Kingdom, with extensive clinical experience in PPCC-care. The diagnostic process to confirm PPCC is not elaborated on in this short communication, but has been described in detail by Sansone et al.^26^ Due to the large impact that PPCC can have on daily life, it is essential that real-time experiences are shared with the international community to exchange and compare treatment regimens worldwide. The previously described studies have found comparable results and recommendations about how to approach and manage children with PPCC, strengthening our results.

## Individual Treatment Plan

**FIGURE 1.**
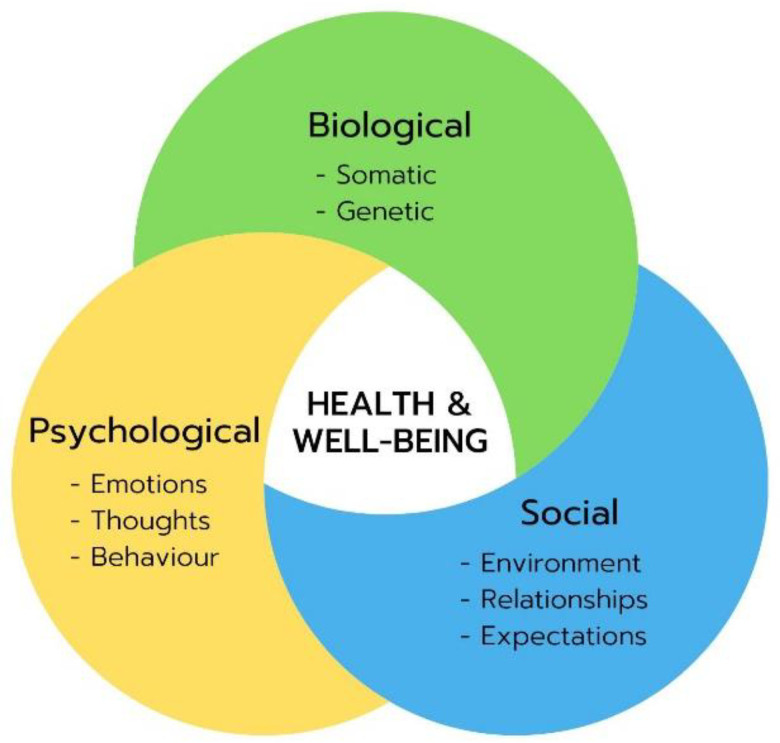
Biopsychosocial model.

The symptoms of PPCC are very heterogeneous. Because of the current lack of understanding of etiologic factors for PPCC, treatment is aimed at symptomatic aspects and energy management. We therefore advise the use of a biopsychosocial approach,^27^ considering physical functioning in the context of biologic (somatic), psychologic (emotions, thoughts, behavior) and social (expectations, environment) aspects (Fig. 1). The contribution and interaction of all three domains differ between children and should thus be addressed individually, preferably by multidisciplinary assessment.

## Coordination and Location of Care

Care should be provided by a multidisciplinary team of primary healthcare professionals, which includes pediatric physiotherapists, occupational therapists, and psychology specialists, led by a general practitioner and/or pediatrician. These professionals focus on improving physical functioning and assist in dividing energy and activities throughout the day and week. For specific nutritional or smell- and taste problems, children are referred to a dietician. For problems with swallowing, children are referred to speech- and language therapists.

Indication and timing of referral to more specialized or secondary care is dependent on the complexity of the pathophysiology and the interactions between the primary treatment response and relevant biopsychosocial factors.

The described multidisciplinary approach can potentially be challenging when children and parents have limited access to care, or when communication between healthcare professionals is lacking. To facilitate multidisciplinary care, we suggest to have regular (online) meetings between healthcare professionals, led by the general practitioner or the pediatrician. Ideally, to optimize management and rehabilitation for children and parents, all care would be centralized within specialized PPCC multidisciplinary clinics, working together with general clinics in shared care constructions. These clinics have been established in for instance the United States^28^ and Isreal,^29^ but are, unfortunately, not generally available for children with PPCC.

## Assessment of (Daily) Functioning

Assessment of daily functioning can be done according to the biopsychosocial approach.^27^ It is important to have a detailed assessment of the child’s energy balance. Certain symptoms, such as extreme fatigue, increased heart rate during or after physical activities, postexertional malaise (PEM), postural orthostatic tachycardia syndrome, dyspnea and mental health symptoms, can be evaluated more closely with validated and reliable tools. Specific questionnaires and measurements that can be helpful in the assessment of daily functioning and participation are described in Table 1.

**TABLE 1.** Questionnaires and Clinimetrics for Overall Assessment of Functioning

Biological	Psychological	Social
To assess physical functioning: - EQ5D family—“Physical functioning”*	To evaluate health-related quality of life: Pediatric quality of life (PedsQL)^30^ - Patient reported, age 5 to 18 years - Parent-reported, age 2 to 18 years	To measure and evaluate wishes and needs of the child and their family: - Canadian Occupational Performance Measure^31^
To assess fatigue: - Pediatric quality of life (PedsQL) Multidimensional fatigue scale* ^32^ - Patient Reported Outcomes Measurement Information System (PROMIS), pediatric fatigue^33^	In case of neurocognitive complaints: - PedsQL cognitive functioning scale* - Neuropsychological assessment by a neuropsychologist	To formulate and evaluate goals: - Goal attainment scaling (GAS)^34^
To monitor (im)balance of activity and rest: - Activity diary - Activity calculator^35^ - Activity tracker (preferably with heartrate monitor)	To evaluate anxiety or depression: - Pediatric PROMIS anxiety^36^ - Pediatric PROMIS depressive symptoms^36^	
To evaluate postural orthostatic tachycardia syndrome (POTS): - The standing test^37^		
To evaluate postexertional malaise (PEM): - DSQ-PEM (only available in Dutch)^38^		
To evaluate dysfunctional breathing or dyspnea: - The Nijmegen questionnaire^39^		
To assess gastrointestinal symptoms: - PedsQL gastrointestinal symptoms scale*		
*Only in case of mild physical complaints, or when children are close to returning to sports, evaluate functional strength and/or exercise (in)tolerance* †		
To measure functional strength: - Bruininks–Oseretsky test of motor proficiency Second Edition—subscale strength (BOT-2)^40^ - Functional strength measurement (FSM)^41^ → Consider adding a heartrate monitor. To evaluate exercise (in)tolerance: - Shuttle run test^42^ - Bruce treadmill test^43^ - Fitkids treadmill test^44^ - Muscle power sprint test (for anaerobic capacity)^45^		

* Measurement was included in a Core Outcome Set for PPCC, developed through a Delphi consensus process by the PC-COS Children project, by Seylanova et al.^46^

† Beware of overexertion, as this can lead to PEM. Also beware of complaints of POTS.^37^

## Symptom Management

As described earlier, PPCC is a heterogeneous illness, with a wide range of symptoms within multiple organ systems.^12^ It is important to address, investigate and manage all symptoms with equal concern, as described in a 15-minute consultation by Wacks et al.^37^

The most prevalent complaint in children with PPCC is fatigue,^15^ often combined with PEM, which is defined as an exacerbation of symptoms (such as muscle weakness or excessive and unjustified fatigue) following exertion.^47^ PEM is hypothesized to be associated with pathophysiological abnormalities in skeletal muscle,^48^ which might be in combination with autonomic dysfunction (ie, hyperactive sympathetic signaling and hypoactive parasympathetic signaling),^49^ and could be a result of an imbalance between daily activities and rest. Management of these symptoms is described below. In addition, the World Health Organization, in collaboration with medical professionals from various countries, has developed a leaflet on self-management of long COVID in adolescents, which addresses multiple strategies for self-managing (balancing) activities, mental health, sleeping, nutrition, pain and education.^50^

## Main Cornerstones of Treatment

Because the underlying pathophysiologic mechanisms of PPCC are still unclear, there are no causal treatment strategies available yet. As the search for underlying mechanisms and biomedical treatment options continues, we will have to rely on treatment that focuses on managing symptoms and a phased return to activities.

It is important to develop a treatment plan together with children and their parents, based on the findings during the initial assessment, using a holistic, individualized, biopsychosocial approach. The main cornerstones of treatment are described below.

### Education About PPCC and the Symptoms That Children Are Experiencing

Validate the child’s and parents experience. Because of the lack of knowledge among healthcare professionals on PPCC, symptoms are often not recognized and/or not taken seriously. By explaining the symptoms and impact on daily life, children and their parents may more easily feel reassured and understood. Provide information about support options from patient representative organizations (eg, LongCovidKids^51^), and provide insight into (all bio, psycho and social) treatment options, with the connotation that these are mostly based on experience and that we don’t have all the answers (yet).^52^

### Energy Management: Finding Balance Between Activities and Moments of Rest

As mentioned before, fatigue and PEM are common and often debilitating symptoms in PPCC. It is therefore crucial to help make children (and their caregivers) aware of their own physical and mental boundaries and establish their personal baseline level of activities. The next step is to educate the child on how to respond appropriately and not cross their boundaries. It is important that children and parents learn strategies for spreading energy expenditure during the day, also known as pacing.^53^ Help them by planning, adapting and choosing activities, which include both physical and cognitive activities. The activity calculator^35^ might be helpful in this process.

An important notion to make is that, although often applied, pacing has not been proven through clinical trials to be effective as a treatment strategy for PPCC. However, a recent review by Sanal-Hayes et al^54^ on pacing as management for myalgic encephalomyelitis/chronic fatigue syndrome (ME/CFS) in adults, showed that pacing was self-reported to be the most efficacious, safe, acceptable, and preferred form of activity management for people with ME/CFS. It should be noted that although many studies reported improvement of chronic fatigue symptoms after pacing, some found no effects, and one reported worsening of symptoms in 14% of their study population. Because some presentations of PPCC are similar to ME/CFS presentations, treatment strategies that seem largely beneficial for ME/CFS, such as pacing, can possibly be helpful in managing post-COVID-19 symptoms.^53^ However, it is important to continuously evaluate the effects of pacing per PPCC-patient, and adjust the activity versus rest schedule where necessary, providing personalized care for each child.

### Return to Daily Life Activities and Improving Physical and Cognitive Functioning

Focus on improving participation in daily life activities (eg, school and social activities), based on goals that are relevant and realistic. A goal-oriented approach to build up activities is more suitable than a time-oriented approach. Focus on activities that are enjoyable for the child, as this can differ greatly between children. Maintaining (light) physical activities seems helpful; therefore, stimulate light physical activity for 20 minutes a day, such as going for a walk. Start physical aerobic training or anaerobic training only if reintegration towards sports has already started, and only when there are no more signs of PEM.

Address sleep disturbances through environmental and behavioral interventions to improve sleep hygiene. Address dietary intake with adequate hydration and eating healthy and regular meals, and involve the dietician if necessary. In case of dysfunctional breathing, it can be helpful to teach breathing exercises or request a consult with a respiratory physiotherapist or pediatric speech therapist.

Return to school is an important goal for many children, but this can be challenging as schools are not always informed about PPCC or the need for altered school hours and slow but steady improvement of daily activities. It is therefore crucial that schools are educated appropriately about PPCC, treatment advice and prognosis,^50^ and are involved in setting up a phased return-to-school plan.

### Addressing Cognitive Complaints and Psychosocial Predisposing Factors

Cognitive complaints such as attention deficit and “brain fog” are common symptoms in children with PPCC.^14^ Cognitive symptoms could be a direct consequence of SARS-CoV-2 infection, hypothesized to be driven by direct and indirect injury to the neural networks.^55^ At the same time, cognitive symptoms can arise secondary to the overall distress and emotional upheaval that typically accompany PPCC and its prolonged, disabling symptoms. It is therefore important to address the personal situation, medical history, and personality of the child, to then develop an individualized treatment plan. In case of anxiety or depressive symptoms, children should be referred to a child psychologist or child psychiatrist.

### Addressing Trials for Specific Complaints and Treatment Strategies

Currently, many trials concerning specific COVID-19-related symptoms are ongoing. Physicians have been prescribing drugs for COVID-19-related symptoms which are like other diagnoses and their symptoms, most likely based on a likewise pathophysiology, such as in postural orthostatic tachycardia syndrome. Therapies currently being offered by some centers include treatment with hyperbaric oxygen^56^ and treatment of microclots.^57^ However, aside from respiratory training, there is little convincing evidence for many prescribed therapeutics,^58^ and they are not (yet) recommended as standard treatments. This poses a dilemma for healthcare professionals: the lack of evidence-based treatment regiments versus the severe need for help of our patients with PPCC. Therefore, it is important to include patients in randomized controlled trials and to inform them about ongoing trials and expected results.

## Follow-up

Regular follow-up of children with PPCC is needed to monitor the process of recovery, preferably using standardized questionnaires and clinimetrics as described in Table 1. Recovery can take time, and in some children, we observed relapses and periods of illness other than COVID-19. During follow-up, reconsider the treatment regime and be aware of complicating factors that require further treatment. Standardized follow-up can facilitate analysis of the long-term functioning of children after COVID-19 and more directed treatment regimes.

## CONCLUSION

Pediatric post-COVID-19 condition is a heterogeneous syndrome and can have a big impact on neurocognitive, psychosocial, and physical functioning, leading to decreased societal participation and affecting school attendance. Scientific evidence on management, rehabilitation, and treatment strategies for children is scarce, and treatment based on pathophysiology does not yet exist. Therefore, we developed an experience-based clinical guideline with treatment options for PPCC. We propose an individualized, multidisciplinary management strategy, with a holistic approach based on the biopsychosocial model, where the main goal should be a return to cognitive, physical, and social activity. To enable pathophysiological-based treatment regimens, we want to highlight the need for prospective (randomized) trials on pathophysiology and treatment strategies for PPCC.
